# Bat Rabies in Massachusetts, USA, 1985–2009

**DOI:** 10.3201/eid1608.100205

**Published:** 2010-08

**Authors:** Xingtai Wang, Alfred DeMaria, Sandra Smole, Catherine M. Brown, Linda Han

**Affiliations:** Massachusetts Department of Public Health, Boston, Massachusetts, USA

**Keywords:** Rabies, bats, exposure, raccoon rabies virus variant, viruses, Massachusetts, dispatch

## Abstract

To investigate rabies in Massachusetts, we analyzed bat rabies test results before and after introduction of raccoon variant rabies and after release of revised 1999 US Advisory Committee on Immunization Practices recommendations for rabies postexposure prophylaxis. Bat submissions were associated with level of rabies awareness and specific postexposure recommendations.

For the past 20 years, bat-associated rabies virus variants have accounted for most human rabies infections acquired in the United States. Most infections were associated with reports of minimal, if any, direct contact with a bat, which suggested that superficial or unrecognized bat bites may transmit infection (*1**–**5*). In 1999, the Advisory Committee on Immunization Practices (ACIP) updated their human rabies prevention guidelines to include consideration of rabies postexposure prophylaxis (PEP) in some circumstances without recognized direct bat contact (*6*). Since the first rabid bat was reported in Massachusetts in 1961, testing of rabid animals has been conducted at the state public health laboratory (*7*). Raccoon rabies virus variant (RRV) was first detected in Massachusetts in 1992 and had spread throughout most of the state by 1996.

We reviewed bat rabies data for Massachusetts during 1985–2009. We analyzed the effect of RRV introduction on specimen submission, the impact of the 1999 ACIP guidelines on specimen submission, and differences in the likelihood of rabies infection among bats with different submission characteristics.

## The Study

**Figure 1:**
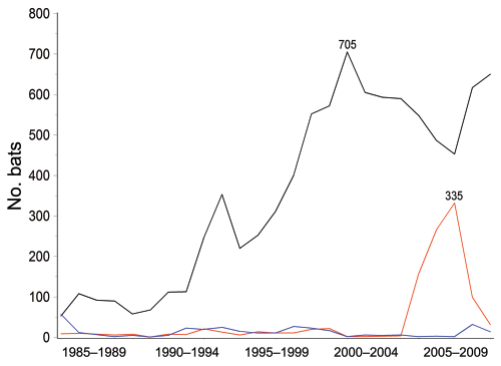
Bats submitted for rabies testing in Massachusetts, USA, 1985–2009. Black line indicates *Eptesicus fuscus*, red line indicates *Myotis lucifugus*, and blue line indicates other pooled bats.

From 1985 through 2009, a total of 10,257 bats were submitted to the Massachusetts Department of Public Health for rabies testing: 8,850 big brown bats (*Eptesicus fuscus*, 86.3%), 1,074 little brown bats (*Myotis lucifugus*, 10.5%), 94 Keen long-eared bats (*Myotis keenii*, 0.9%), 48 red bats (*Lasiurus blossevillii*, 0.5%), 17 hoary bats (*Lasiurus cinereus*, 0.2%), 17 silver-haired bats (*Lasionycteris noctivagans*, 0.2%), 1 zoo-submitted Seychelles fruit bat (*Pteropus seychellensis*), and 156 (1.5%) unspeciated bats. The proportion of rabies-positive bats by species was 5.0% (443/8,850) big brown bats, 3.6% (39/1,074) little brown bats, 23.5% (4/17) hoary bats, 8.3% (4/48) red bats, 5.9% (1/17) silver-haired bats, and 3.2% (3/94) Keen long-eared bats; 751 (7.3%) of 10,257 bats were not suitable for rabies testing. Among all rabies-positive bats, 89.3% were big brown bats, 8.0% were little brown bats, and 2.7% were of less frequently submitted species. During 2005–2008 (Figure 1), submissions of little brown bat sharply increased then decreased, although the proportion that was rabies positive (2%–4%) remained stable.

**Figure 2:**
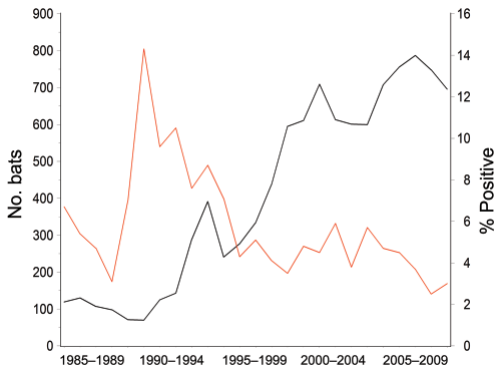
Rabies in bats in Massachusetts, USA, 1985–2009. Black line indicates number of bats submitted and red line indicates percentage of bats positive for rabies.

The average annual number of bat submissions increased significantly from 103 during 1985–1991 to 302 during 1992–1998 and to 675 during 1999–2009 (p<0.0001) (Figure 2). The average annual number of confirmed rabid bats increased from 7 to 19 to 28 for those periods, and the proportion of bats positive for rabies decreased from 6.9% (50/720) to 6.4% (135/2,113) to 4.2% (311/7,424). The proportion of rabid bats was significantly lower during 1999–2009 (p<0.05).

Among 961 testable bats submitted before RRV introduction, 76 (7.9%) were positive for rabies compared with 420 (4.9%) of 8,545 bats submitted after RRV introduction. No positive association was identified between RRV introduction and proportion of bats positive for rabies, even when adjusting for potential confounders such as bat species (big brown and little brown vs. other pooled species), reason for bat submission (human and pet exposure, human exposure only, and pet exposure only vs. undefined), and time of submission (1985–1991 and 1992–1998 vs. 1999–2009). Limited RRV strain typing results performed on 52 rabies-positive bats showed that all bats were infected with non-RRV (X. Wang et al., unpub. data).

Before publication of the 1999 ACIP recommendations, the most common reason for testing bats was pet exposure only, which accounted for 50.3% of submissions during 1985–1991 and 43.5% during 1992–1998 (Table 1). After 1999, the most common reason for bat testing was human exposure, accounting for 72.0% of submissions during 1999–2009. Although the number of bat submissions because of human exposure increased with time, the rabies-positive proportion of these bats decreased from 10.3% (17/165) to 5.8% (51/885) to 3.8% (204/5,343) during the 3 periods. No significant differences in rabies positivity among bats submitted for different reasons were found during 1999–2009 (Table 2).

**Table 1:** Reasons for bat submissions for rabies testing, Massachusetts, USA, 1985–2009

Reason	No. (%) submissions		
	1985–1991	1992–1998	1999–2009
Human and pet exposure	5 (0.7)	250 (11.8)	1,004 (13.5)
Human exposure only	160 (22.2)	635 (30.1)	4,339 (58.5)
Pet exposure only	362 (50.3)	918 (43.5)	1,295 (17.4)
Sickness and other reasons	193 (26.8)	310 (14.7)	786 (10.6)

Total

720

2,113

7,424

**Table 2:** Characteristics of bats submitted for rabies testing, Massachusetts, USA, 1985–2009*

Characteristic	No. rabid/no. tested (%)		
	1985–1991	1992–1998	1999–2009
Total no. bats	50/720 (6.9)	135/2,113 (6.4)	311/7,424 (4.2)
Bat species			
Big brown (*Eptesicus fuscus*)	47/581 (8.1)	126/1,898 (6.6)	270/6,371 (4.2)
Hoary (*Lasiurus cinereus*)	0/5	4/12 (33.3)	NS
Little brown (*Myotis lucifugus*)	1/50 (2.0)	2/83 (2.4)	36/941(3.8)
Keen long-eared (*M. keenii*)	2/9 (22.2)	1/54 (1.9)	0/31
Red (*L. blossevillii*)	0/8	2/20 (10.0)	2/20 (10.0)
Seychelles fruit (*Pteropus seychellensis*)	NS	0/1	NS
Silver-haired (*Lasionycteris noctivagans*)	NS	0/4	1/13 (7.7)
Unidentified	0/67	0/41	2/48 (4.2)
Reasons for bat test			
Human and pet exposure	0/5	16/250 (6.4)	40/1,004 (4.0)
Human exposure	17/160 (10.6)	35/635 (5.5)	164/4,339 (3.8)
Pet exposure	21/362 (5.8)	50/918 (5.5)	67/1,295 (5.2)
Sick and other reasons	12/193 (6.2)	34/310 (11.0)	40/786 (5.1)
Signs			
Aggression			
Unobserved	NC	118/2,033 (5.8)	291/7,229 (4.0)
Yes	NC	17/80 (21.3)	20/196 (10.3)
Ataxia			
Unobserved	NC	128/2,087 (6.1)	307/7,392 (4.2)
Yes	NC	7/26 (26.9)	4/32 (12.5)
Death			
Unobserved	NC	114/1,765 (6.5)	284/6,566 (4.3)
Yes	NC	21/348 (6.0)	27/858 (3.2)
Disorientation			
Unobserved	NC	113/1,985 (5.7)	278/7,038 (4.0)
Yes	NC	22/128 (17.2)	33/386 (8.6)
Lethargy			
Unobserved	NC	119/2,034 (5.9)	275/7,145 (3.9)
Yes	NC	16/79 (20.3)	36/279 (12.9)
Paralysis			
Unobserved	NC	135/2,105 (6.4)	304/7,394 (4.1)
Yes	NC	0/8	7/30 (23.7)
Salivation			
Unobserved	NC	135/2,111 (6.4)	309/7,409 (4.2)
Yes	NC	0/2	2/15 (13.3)
Seizures			
Unobserved	NC	135/2,107 (6.4)	309/7,416 (4.2)
Yes	NC	0/6	2/8 (25.0)

*NS, no specimen; unobserved, none or unknown; NC, not collected.

Clinical signs were reported for 2,291 (24%) of 9,537 bats submitted since 1992 (Table 2). The most common signs were death (1,206, 52.6%), disorientation (514, 22.4%), lethargy (358, 15.6%), or aggressiveness (275, 12.0%). Bats described as having aggression, ataxia, disorientation, or lethargy were significantly more likely to have rabies than were bats with no reported signs (p<0.05). Bats found dead were no more likely to have rabies than were bats reported alive before submission.

## Conclusions

The increase in bat submissions for rabies testing after 1992 correlated with RRV introduction and associated statewide enhancement of rabies surveillance and awareness generated by arrival of raccoon rabies. However, in contrast to other animal species in which RRV introduction resulted in an increase in identification of rabies (*8*), the proportion of bats with rabies was constant in the periods before (1985–1991) and after (1992–1998) RRV introduction. These findings are supported by limited laboratory typing data in rabid bats, which showed no evidence that RRV plays a role in bat infection. This finding is consistent with reports that insectivorous bat rabies virus variants circulate separately from terrestrial viral variants (*9**,**10*). Although incidents of bat rabies virus variant spillover into terrestrial mammals are documented (*11*), spillover of terrestrial variants into bats has yet to be reported.

After release of the ACIP guidelines, the number of annual bat submissions for rabies testing doubled relative to the previous period, and the proportion of rabies-positive bats decreased (6.5% vs. 4.2%). Between 1985–1991 and 1999–2009, the proportion of bats submitted on the basis of human exposure (human exposure alone and human and pet exposure) increased from 22.9% to 72.0%. This finding was likely caused by adherence to the ACIP recommendations and increased awareness of rabies in bats.

In examining the effect of various bat characteristics on the likelihood of rabies infection, we found that signs of central nervous system involvement, including aggression, ataxia, disorientation, or lethargy, were associated with rabies. However, use of reported bat behavior and appearance in assessing the risk for rabies is not feasible (*8*).

Big brown bats were submitted in the highest numbers, and had the highest rabies positivity. Increases in little brown bat submissions began 1 year before identification of bat white-nose syndrome in upstate New York in 2006 (*12*). This increase in little brown bat submissions was not associated with rabies positivity or with bats found dead as a reason for submission.

The role of rabies laboratory testing and public health follow-up is reflected in part by the number of costly courses of PEP potentially averted or discontinued. During 1999–2009, a total of 4,766 Massachusetts residents were exposed to bats that were negative for rabies virus. With each course of PEP costing an estimated $2,376 in biologics alone in 1998 (*13*), and without considering costs associated with medical evaluation and vaccine administration, this cost amounts to $10–20 million in healthcare savings in Massachusetts in 1999–2009. A recent study of PEP recommendations for potentially unrecognized bat exposures suggests that the rate of human rabies associated with such exposures was only 1/2.7 billion person-years, and medical costs of such exposures could be up to 2 billion Canadian dollars (*14*). Analyses such as this have already prompted changes in rabies PEP recommendations in Canada to specify direct exposure to a bat (*15*). PEP recommendations in the United States are based on national guidelines and include considerations of PEP for cryptic bat exposures. Current practice is in place pending reconsideration of and changes to these guidelines.
